# Supplementary material for: S-Methylmethionine Effectively Alleviates Stress in Szarvasi-1 Energy Grass by Reducing Root-to-Shoot Cadmium Translocation
Source: Plants (Basel). 2022 Nov 4;11(21):2979. doi: 10.3390/plants11212979 (PMC9654709; doi:10.3390/plants11212979)
Supplement: Supplementary file 1 [file plants-11-02979-s001.zip › Supplementary_Figures.pdf]

S-methylmethionine effectively alleviates stress in Szarvasi-1 energy grass by reducing root-to-shoot cadmium translocation

Deepali Rana, Vitor Arcoverde Cerveira Sterner, Aravinda Kumar Potluri, Zoltán May, Brigitta Müller , Ádám Solti, Szabolcs Rudnóy, Gyula Sipos, Csaba Gyuricza and Ferenc Fodor

Supplementary Figures

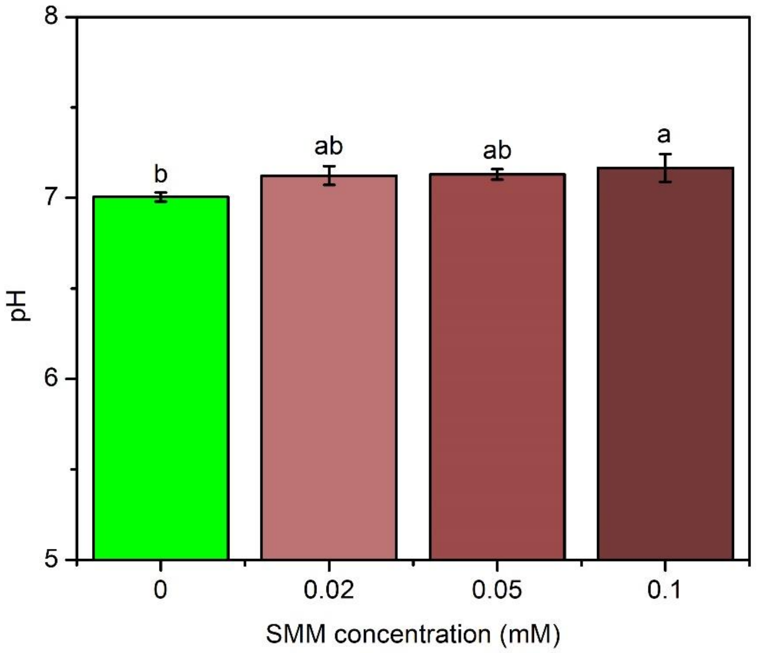

Supplementary Figure S1. pH of the nutrient solution of Szarvasi-1 energy grass one week after a one-day pre-treatment with SMM. Error bars represent SD values. To compare the differences, one-way ANOVA was performed on each dataset combined with Tukey–Kramer post-hoc test on the treatments ($p<0.05$). Different lowercase letters indicate significantly different groups.

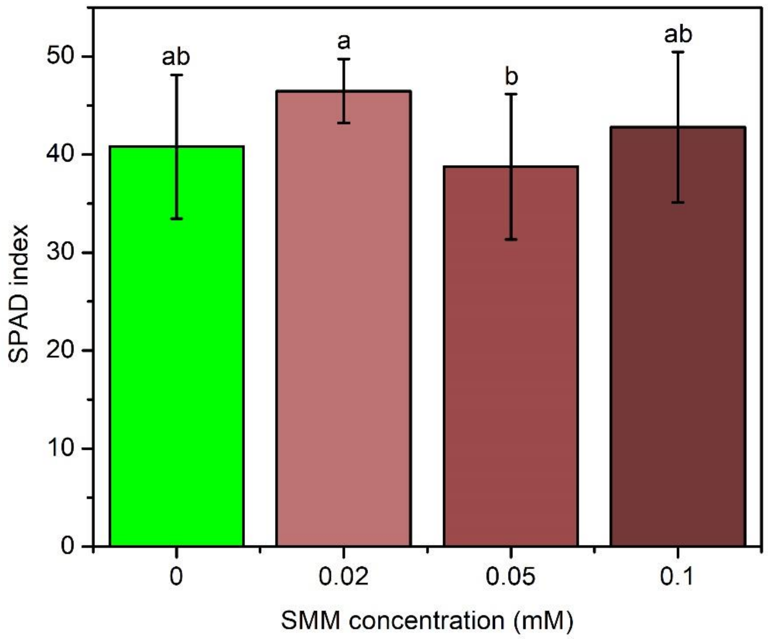

Supplementary Figure S2. Chlorophyll content in the oldest fully developed leaves of Szarvasi-1 energy grass measured as SPAD index one week after a one-day pretreatment with SMM. Error bars represent SD values. To compare the differences, one-way ANOVA was performed on each dataset combined with Tukey-Kramer post–hoc test on the treatments ($p<0.05$). Different lowercase letters indicate significantly different groups.

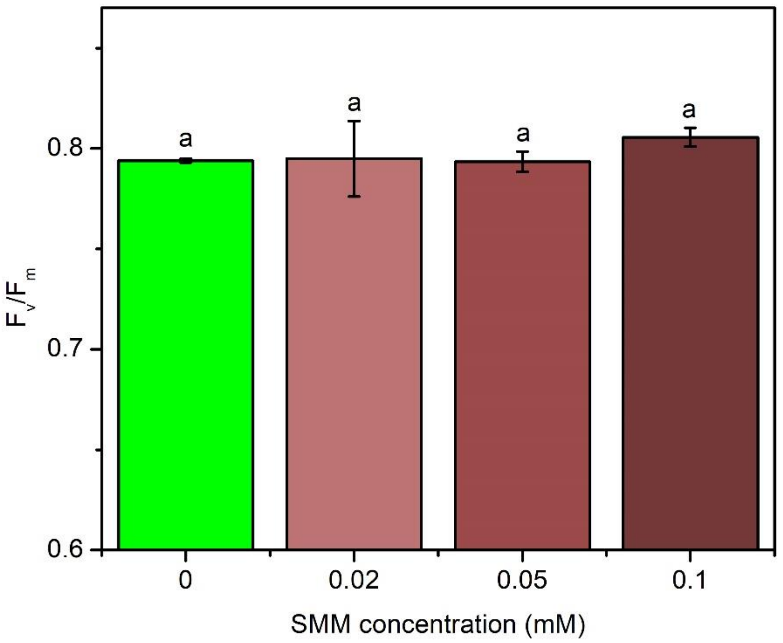

Supplementary Figure S3. Maximal quantum efficiency of Photosystem II reaction centres (F_v/F_m) in the youngest fully developed leaves of Szarvasi-1 energy grass one week after a one-day pretreatment with SMM. Error bars represent SD values. To compare the differences, one-way ANOVA was performed on each dataset combined with Tukey–Kramer post-hoc test on the treatments ($p<0.05$). Different lowercase letters indicate significantly different groups.

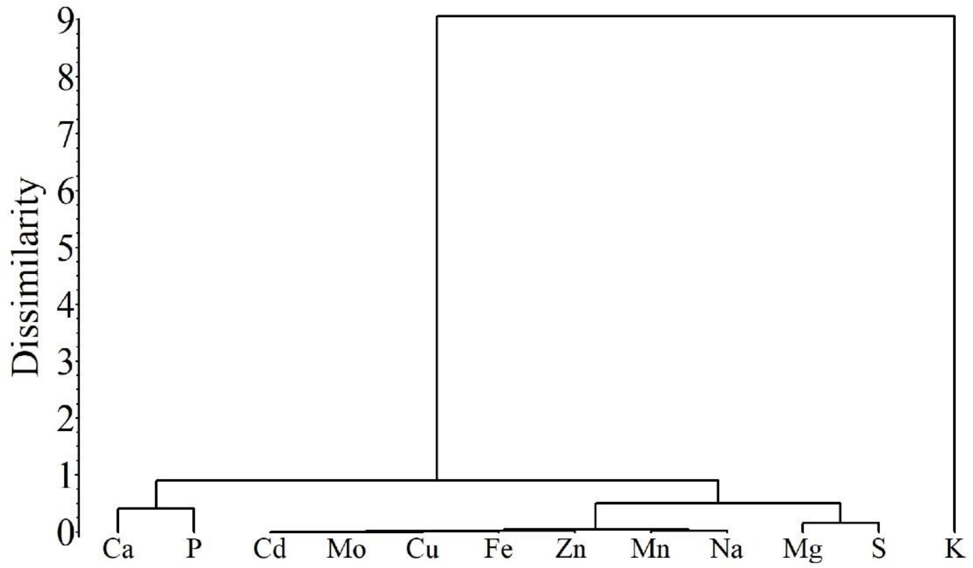

Supplementary Figure S4A. Clustering of Cd and nutrient element uptake pattern in shoot samples of control Szarvasi-1 energy grass plants.

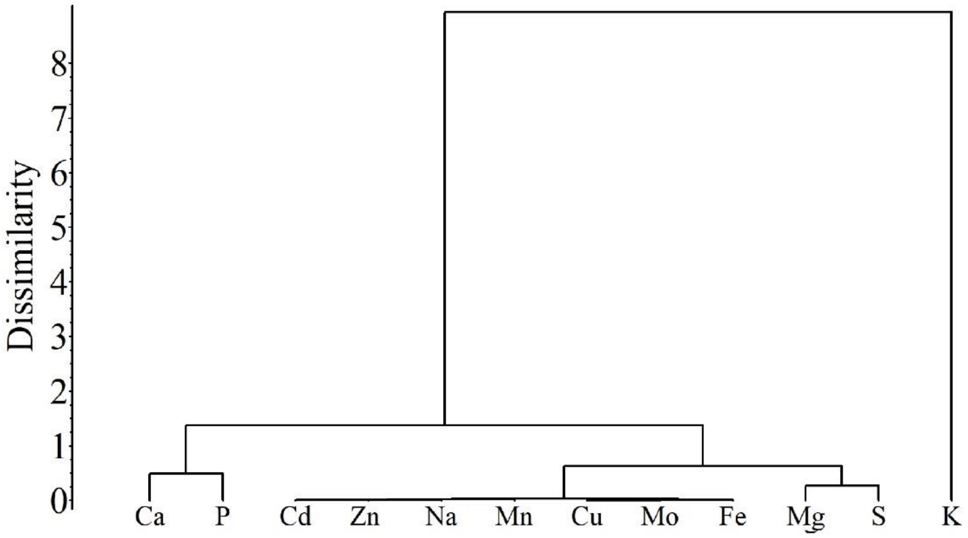

Supplementary Figure S4B. Clustering of Cd and nutrient element uptake pattern in shoot samples of Szarvasi-1 energy grass plants exposed to 0.01 mM Cd for two weeks.

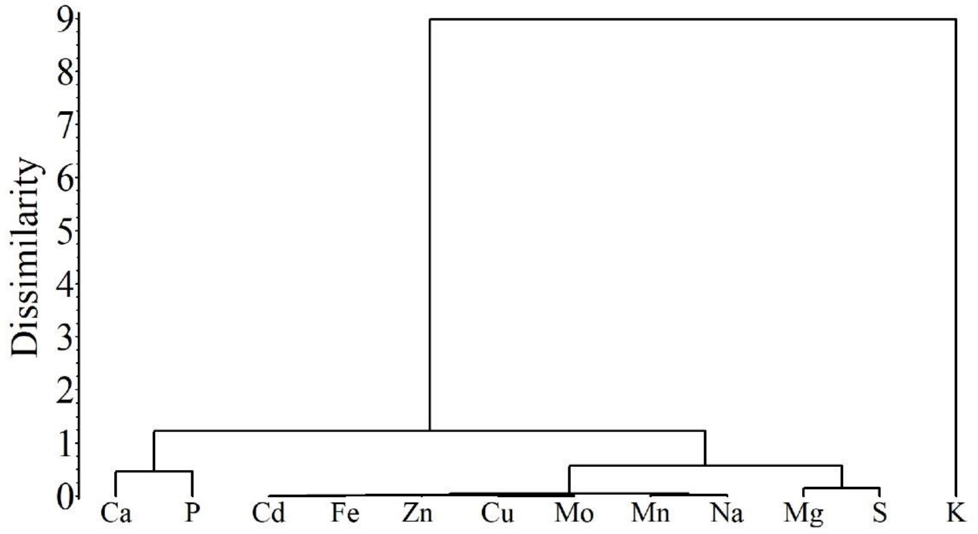

Supplementary Figure S4C. Clustering of Cd and nutrient element uptake pattern in shoot samples of Szarvasi-1 energy grass plants primed with 0.02 mM SMM for one day and then exposed to 0.01 mM Cd for two weeks.

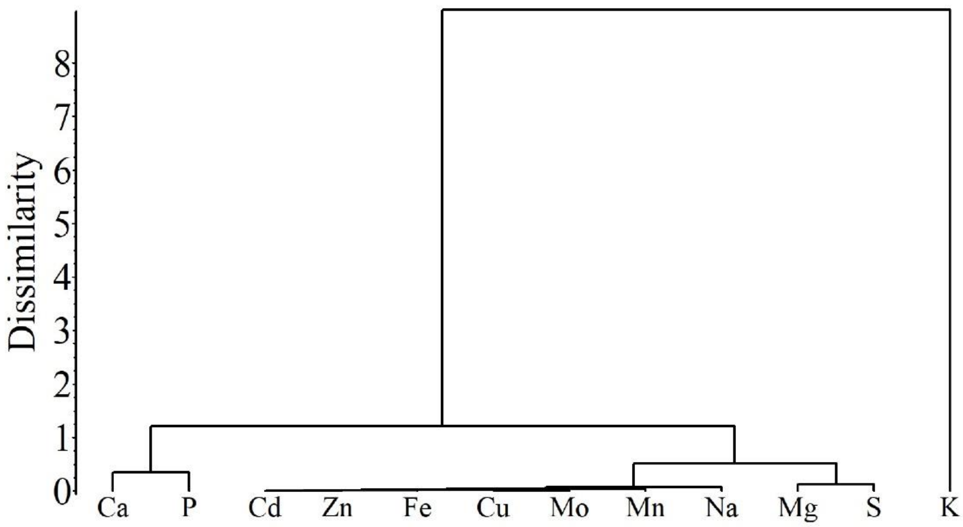

Supplementary Figure S4D. Clustering of Cd and nutrient element uptake pattern in shoot samples of Szarvasi-1 energy grass plants primed with 0.05 mM SMM for one day and then exposed to 0.01 mM Cd for two weeks.

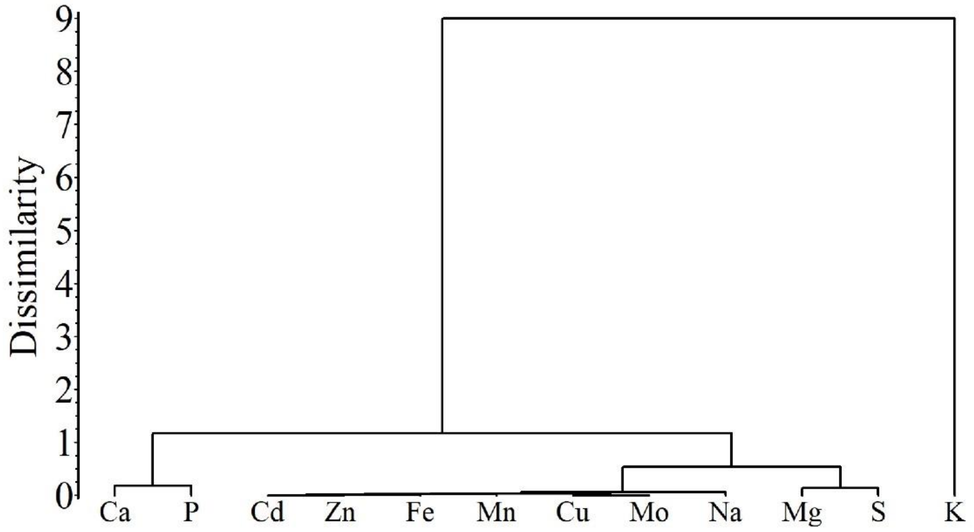

Supplementary Figure S4E. Clustering of Cd and nutrient element uptake pattern in shoot samples of Szarvasi-1 energy grass plants primed with 0.1 mM SMM for one day and then exposed to 0.01 mM Cd for two weeks.

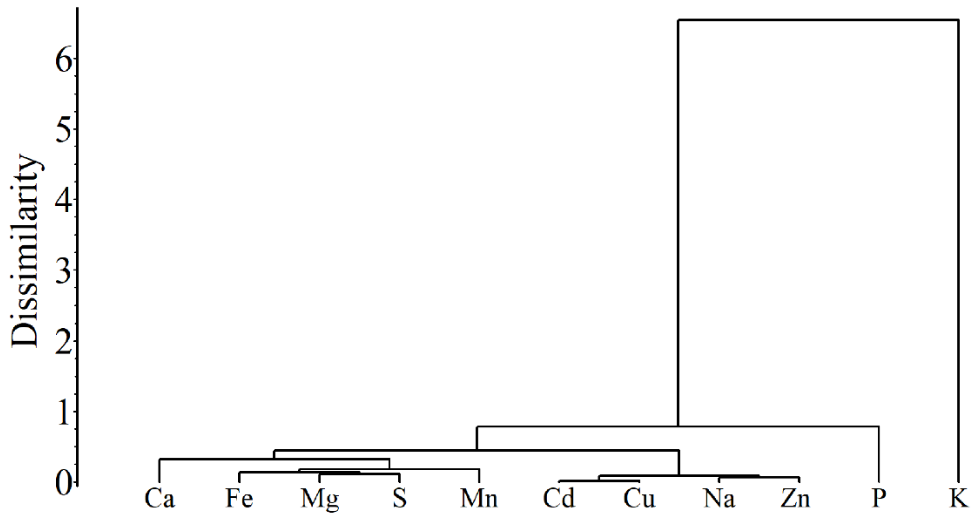

Supplementary Figure S5A. Clustering of Cd and nutrient element uptake pattern in root samples of control Szarvasi-1 energy grass plants.

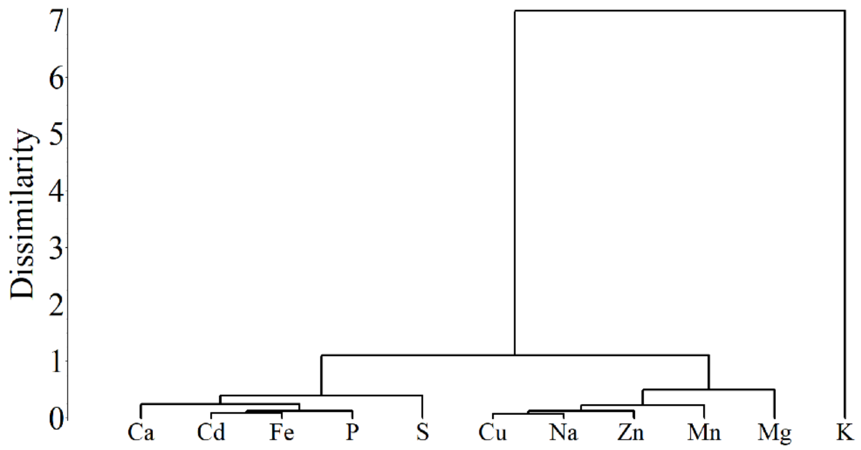

Supplementary Figure S5B. Clustering of Cd and nutrient element uptake pattern in root samples of Szarvasi-1 energy grass plants exposed to 0.01 mM Cd for two weeks.

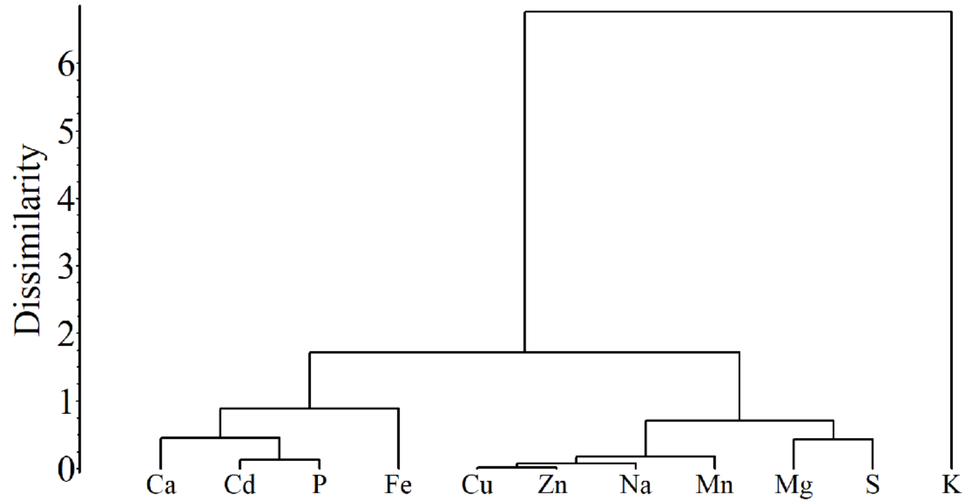

Supplementary Figure S5C. Clustering of Cd and nutrient element uptake pattern in root samples of Szarvasi-1 energy grass plants primed with 0.02 mM SMM for one day and then exposed to 0.01 mM Cd for two weeks.

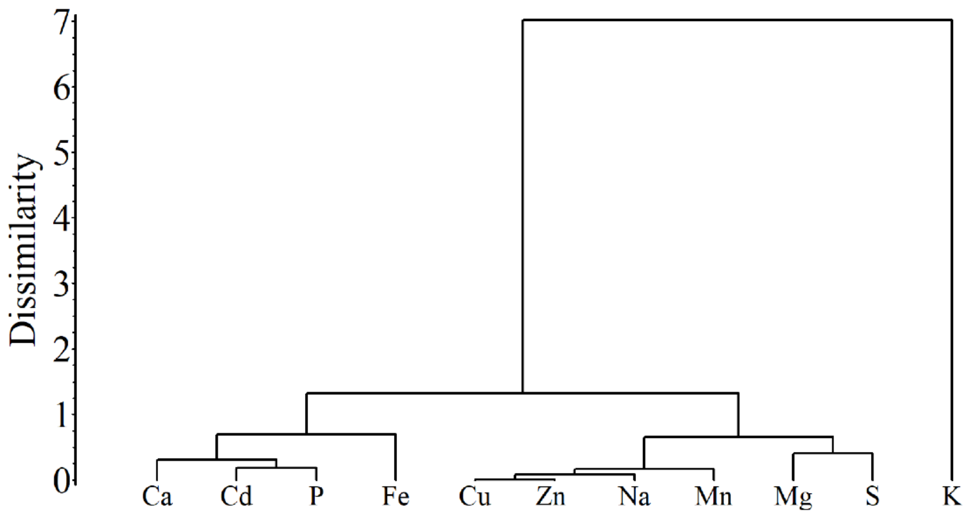

Supplementary Figure S5D. Clustering of Cd and nutrient element uptake pattern in root samples of Szarvasi-1 energy grass plants primed with 0.05 mM SMM for one day and then exposed to 0.01 mM Cd for two weeks.

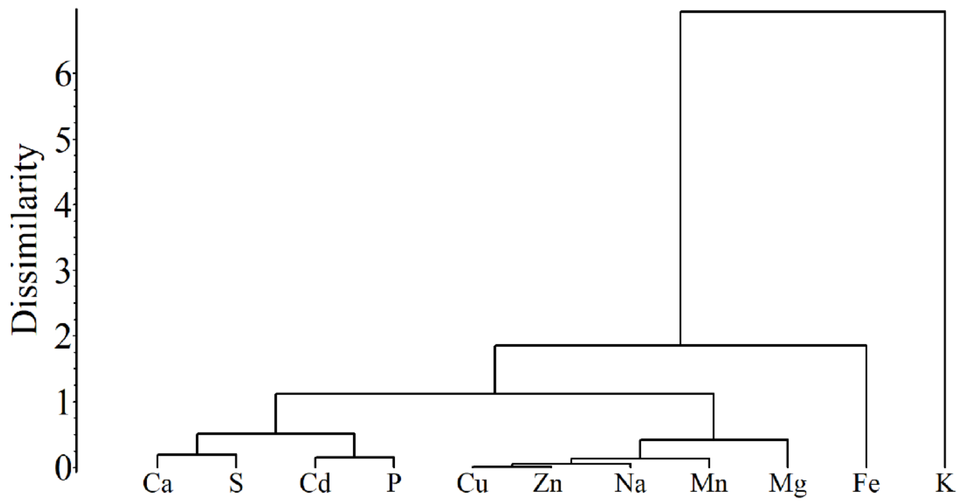

Supplementary Figure S5E. Clustering of Cd and nutrient element uptake pattern in root samples of Szarvasi-1 energy grass plants primed with 0.1 mM SMM for one day and then exposed to 0.01 mM Cd for two weeks.
