# Supplementary material for: S-Methylmethionine Effectively Alleviates Stress in Szarvasi-1 Energy Grass by Reducing Root-to-Shoot Cadmium Translocation
Source: Plants (Basel). 2022 Nov 4;11(21):2979. doi: 10.3390/plants11212979 (PMC9654709; doi:10.3390/plants11212979)
Supplement: Supplementary file 1 [file plants-11-02979-s001.zip › Supplementary_Tables.pdf]

S-methylmethionine effectively alleviates stress in Szarvasi-1 energy grass by reducing root-to-shoot cadmium translocation

Deepali Rana, Vitor Arcoverde Cerveira Sterner, Aravinda Kumar Potluri, Zoltán May, Brigitta Müller , Ádám Solti, Szabolcs Rudnóy, Gyula Sipos, Csaba Gyuricza and Ferenc Fodor

Supplementary Tables

Supplementary Table S1. Number of tillers of Szarvasi-1 energy grass in the pots pre-treated with 0, 0.02, 0.05 and 0.1 mM SMM and exposed to 0 and 0.01 mM Cd. (The initial number of tillers at planting was 3.) To compare the differences, one-way ANOVA was performed on each dataset combined with Tukey–Kramer post-hoc test on the treatments ($p<0.05$). Different lowercase letters indicate significantly different groups.

**Treatment**	**Number of tillers / pot**
Control	4.25 ± 1.50
Cd	3.00 ± 0.00
0.02SMM+Cd	4.00 ± 0.82
0.05SMM+Cd	4.00 ± 0.82
0.1SMM+Cd	4.00 ± 1.00

Supplementary Table S2. Element concentrations in the roots (A) and shoots (B) of Szarvasi-1 energy grass treated with 0, 0.02, 0.05 and 0.1 mM SMM and exposed to 0 and 0.01 mM Cd. To compare the differences, one-way ANOVA was performed on each dataset combined with Tukey–Kramer post-hoc test on the treatments ($p<0.05$). Different lowercase letters indicate significantly different groups.

A

**Element**	**Control**	**Cd**	**0.02SMM+Cd**	**0.05SMM+Cd**	**0.1SMM+Cd**
**Ca**	4283<br>± 840a	4175<br>± 867a	4848<br>± 1281a	3655<br>± 286a	3772<br>± 326a
**K**	41146<br>± 3083a	25982<br>± 7676b	20334<br>± 3886b	22653<br>± 2146b	26335<br>± 2534b
**Mg**	2917<br>± 232a	1954<br>±203b	1557<br>±362b	1579<br>±268b	1716<br>±349b
**P**	6353<br>± 316a	4409<br>± 486c	4621<br>± 602bc	4507<br>± 424bc	5287<br>± 347b
**S**	2312<br>± 260c	3084<br>± 403ab	2605<br>± 194bc	2702<br>± 273abc	3329<br>± 522a
**Cu**	119<br>±12ab	104<br>±23b	94<br>±12bc	108<br>± 42bc	171<br>± 36a
**Fe**	3032<br>± 644c	4407<br>± 332bc	6804<br>± 1314a	6060<br>± 1116ab	8199<br>± 1751a
**Mn**	2095<br>± 711a	828<br>± 251b	624<br>± 87b	552<br>± 212b	596<br>± 237b
**Na**	471<br>±185a	353<br>± 179a	299<br>± 94a	337<br>± 134a	340<br>± 64a
**Zn**	731<br>± 153a	512<br>± 53b	120<br>± 48c	118<br>± 48c	163<br>± 38c

The element concentrations (mean±SD) are expressed in $\mu g\ g^{-1}$.

Supplementary Table S2. Element concentrations in the roots (A) and shoots (B) of Szarvasi-1 energy grass treated with 0, 0.02, 0.05 and 0.1 mM SMM and exposed to 0 and 0.01 mM Cd. To compare the differences, one-way ANOVA was performed on each dataset combined with Tukey–Kramer post-hoc test on the treatments ($p<0.05$). Different lowercase letters indicate significantly different groups.

B

**Element**	**Control**	**Cd**	**0.02SMM+Cd**	**0.05SMM+Cd**	**0.1SMM+Cd**
**Ca**	4093.11 ± 1649.62a	5844.45 ± 1372.00a	4580.93 ± 1504.73a	4700.89 ± 1370.80a	5038.86 ± 943.17a
**K**	44322.95 ± 4051.17a	33126.59 ± 3186.24bc	28551.23 ± 2620.17c	31692.08 ± 2100.50bc	35533.94 ± 4391.63b
**Mg**	2612.83 ± 614.46ab	2683.80 ±481.44a	2012.30 ± 318.90ab	1989.43 ± 317.00b	2398.38 ± 419.87ab
**P**	5475.68 ± 1107.12a	4797.54 ± 713.41abc	3677.42 ± 503.01c	4206.34 ± 468.18bc	4936.55 ± 772.80ab
**S**	2353.38 ± 379.05a	1867.62 ± 237.38b	1600.11 ± 157.03b	1676.23 ± 153.87b	1884.72 ± 233.93b
**Cu**	12.78 ± 2.02a	6.45 ± 0.53b	6.82 ± 1.53b	6.82 ± 2.19b	7.72 ± 1.68b
**Fe**	62.24 ±9.59ab	45.23 ± 12.71b	64.77 ± 12.30ab	73.10 ± 18.96a	81.07 ± 16.80a
**Mn**	262.30 ±81.68a	196.51 ± 51.13ab	186.09 ± 38.64ab	150.85 ± 47.47b	197.39 ± 39.13ab
**Mo**	4.16 ± 1.57a	3.39 ± 0.80a	0.80 ± 0.27b	0.93 ± 0.42b	1.38 ± 0.44b
**Na**	215.94 ± 86.38a	142.51 ±26.80a	190.51 ± 84.01a	271.00 ± 161.38a	263.81 ± 136.63a
**Zn**	138.82 ± 29.17a	100.07 ± 25.04b	82.06 ± 11.36b	69.69 ± 18.19b	93.92 ± 10.06b

The element concentrations (mean±SD) are expressed in $\mu g\ g^{-1}$.
